# From virtual to reality: application of a novel 3D printing hollow model for early-stage lung cancer in the clinical teaching of thoracoscopic sublobar resection

**DOI:** 10.3389/fonc.2025.1526592

**Published:** 2025-05-27

**Authors:** Di Yang, Yafei Miao, Linqian Li, Qing Yu, Qiang Guo, Hefei Li, Xuguang Zhang, Shujie Cheng, Jinghua Li, Ke Zhang

**Affiliations:** ^1^ Clinical Medical College of Hebei University, Affiliated Hospital of Hebei University, Baoding, China; ^2^ Thoracic Surgery Department, Affiliated Hospital of Hebei University, Baoding, China; ^3^ Institute of Life Science and Green Development, Hebei University, Baoding, China; ^4^ Basic Research Key Laboratory of General Surgery for Digital Medicine, Affiliated Hospital of Hebei University, Baoding, Hebei, China; ^5^ 3D Image and 3D Printing Center, Affiliated Hospital of Hebei University, Baoding, Hebei, China; ^6^ Surgical Department, Affiliated Hospital of Hebei University, Baoding, Hebei, China

**Keywords:** medical education, clinical teaching, 3D visualization, 3D printing, sublobar resection

## Abstract

**Background:**

The integration of medical-engineering interdisciplinary technology has transformed clinical skills and anatomical knowledge teaching. Three-dimensional printing (3DP), an innovative tool, shows promise in enhancing surgical training and anatomical understanding. This study evaluates the educational efficacy of a 3DP lung cancer model optimized for surgery in teaching thoracoscopic sublobar resection.

**Methods:**

A total of 62 clinical interns were randomly assigned into two groups: a 3D visualization (3DV) model group and a 3DP model group. Pre- and post-teaching test scores were compared to assess the effectiveness of both models in enhancing anatomical knowledge and surgical skills. Additionally, feedback was collected from the interns regarding the advantages of each model.

**Results:**

There was no significant difference in the pre-teaching test scores between the two groups (*P* > 0.05). However, post-teaching scores in the 3DP group were significantly higher than those in the 3DV group (*P* < 0.05). Survey feedback revealed that the 3DV group excelled in convenience (*P* < 0.001), while the 3DP group demonstrated superiority in the ease of knowledge acquisition and understanding of vascular spatial relationships (*P* < 0.001). No significant differences were found between the two groups regarding model intuitiveness and identification of the lung segment range influenced by the safety margin (*P* > 0.05).

**Conclusion:**

The 3DP model, featuring a transparent hollow sublobar boundary, significantly improved comprehension of complex anatomical relationships and enhanced teaching outcomes in surgical skills. It offers an innovative and effective tool for teaching thoracoscopic sublobar resection, with potential applications in surgical navigation.

## Introduction

The application of advanced 3D technology in medical education is driving significant changes in the teaching of clinical skills and anatomical knowledge. 3D printing (3DP), also known as additive manufacturing, is a technique that creates 3D objects by layering materials (1). Currently, 3DP has been widely applied in various fields, including preoperative planning and medical education (2–5). Based on individual patient imaging data, such as CT or MRI, 3DP can produce highly realistic anatomical models (6). These models not only closely resemble actual anatomical structures but also provide a hands-on, 3D visual and tactile experience, compensating for the limitations of traditional 2D images in terms of intuitiveness and interactive learning. They meet the need for students to develop a tactile and spatial understanding of human anatomy (7).

However, there are some limitations in current 3DP models used in clinical practice, such as refractive errors due to transparent parenchyma, a lack of information on the safe margins of lesions, unclear sublobar boundaries, and obstructed visualization of surgery-related structures. These issues significantly affect the effectiveness of teaching. We believe that for 3DP models to fully realize their potential in clinical teaching and surgery, substantial optimization tailored to surgical needs is required.

Traditional clinical teaching has typically relied on cadaver dissection or 2D imaging (8, 9). While these methods are effective, they have many limitations. For example, cadaver resources are scarce, and preservation and handling are complex. Furthermore, due to the opacity of lung parenchyma, it is difficult to teach sublobar structures within the lungs. Meanwhile, 2D images and anatomical atlases lack three-dimensionality, making it harder to understand the spatial relationships of anatomical structures. Even 3DV digital models require students to mentally construct the 3D information of tissues from interacting with a 2D screen, rather than directly acquiring 3D information. In contrast, 3DP models provide a tangible and innovative teaching tool that can accurately recreate specific anatomical structures. The display of vascular and tumor relationships within organs is especially intuitive, and each model can be customized according to teaching needs. The combined visual and tactile feedback allows students to efficiently learn and practice in a realistic simulation environment, yielding better educational outcomes than traditional imaging and anatomical atlases.

In video-assisted thoracoscopic sublobar resection, precise anatomical information is critical. Due to its minimally invasive nature, the procedure requires an in-depth understanding of lung anatomy, particularly pulmonary vessel variations and resection boundaries, along with precise surgical execution. This not only demands high levels of skill from clinicians but also adds complexity to clinical teaching. A 3DP model can clearly display the 3D relationships between vessels, bronchi, tumors, and sublobar structures, which are essential for accurate VATS sublobar resection (10). Understanding these relationships plays a key role in performing this procedure effectively, and it enhances both clinicians’ and students’ comprehension of anatomy, physiology, tumor characteristics, and surgical techniques. The optimized 3DP model also provides users with more surgical elements and facilitates faster acquisition of vital information.

In this study, we developed a more advanced and innovative 3DP model. The aim of this research is to evaluate the effectiveness of the model in clinical teaching, particularly in enhancing anatomical knowledge and surgical skills. We also gathered feedback from interns regarding the model’s key advantages, providing scientific evidence and practical guidance for the model’s future applications in surgical navigation and medical education.

## Materials and methods

### Participants and grouping

The study included 62 fourth-year clinical medicine undergraduate students who completed their internship at Hebei University Affiliated Hospital from January 2023 to January 2024. The participants were randomly assigned to two groups, the 3DV group and the 3DP group, using a computer-generated random number table. The inclusion criteria were completion of basic medical courses (such as Human Anatomy and Physiology) and certain clinical courses (such as Internal Medicine and Surgery), which provided students with a foundational understanding of human structure and function, thereby offering theoretical support for understanding the anatomical and functional relationships of 3D models. The exclusion criteria were (1): absenteeism (2); previously participated in other educational research involving lung 3DP models or educational research using 3DP models developed by our institution. The study was approved by the Ethics Committee of Hebei University Affiliated Hospital (HDFYLL-KY-2024-134) All participants provided written informed consent and voluntarily participated in the study, with the right to withdraw at any time. Participants’ privacy and autonomy were strictly protected throughout the research process.

### Sample size calculation

Under the guidance of a statistician, the sample size was estimated using G*Power software (Version 3.1.9.7, Dusseldorf, Germany). Based on an effect size (d) of 0.8, a significance level (α) of 0.05, and a statistical power (1-β) of 0.80, the required sample size was calculated to be 52 interns. Considering the actual number of available students, a total of 62 participants were recruited. These participants were randomly assigned to one of two groups using a computer-generated random number table, with each group receiving a different teaching method.

### 3D reconstruction and model 3D printing

**Figure 1:**
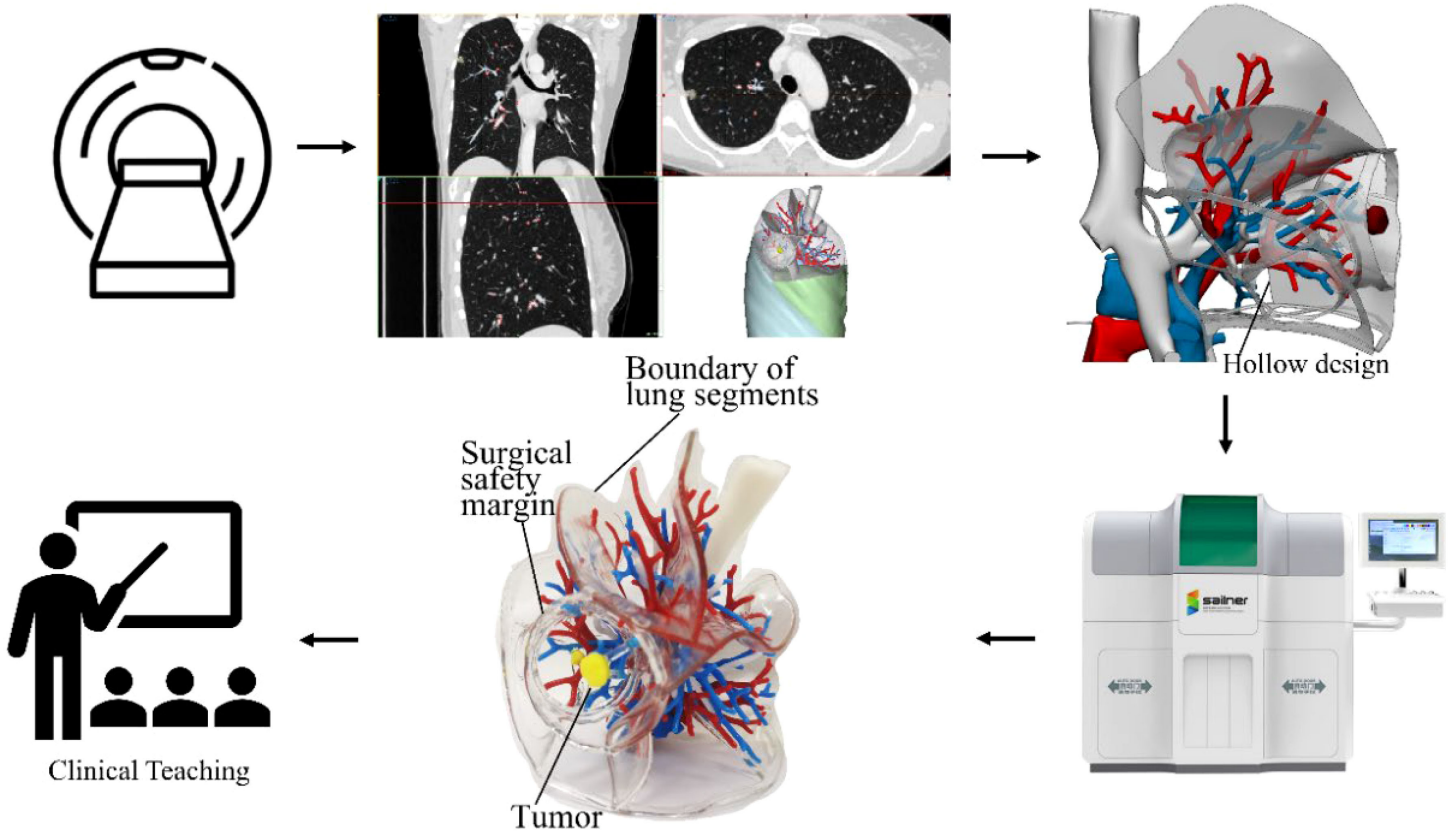
Flowchart of the study process. The figure illustrates the workflow from CT images of a right upper lobe lung cancer case to 3D reconstruction, followed by the printing of a physical model, and finally its application in clinical teaching. After innovative hollowing of the root, both the safety margin and the lung segment boundary have been displayed.

**Figure 1** illustrates the entire process of applying the 3DP model to clinical teaching. Lung CT scan images were imported into Mimics 23.0 software (Materialise, Belgium) in DICOM format for initial 3D reconstruction, followed by post-processing using 3-matic 15.0 software (Materialise, Belgium). The 3DV model, which included bronchi, pulmonary arteries, pulmonary veins, tumors, lung lobes, and segments, was reconstructed from the CT scan images. Mimics software was then used to accurately delineate sublobar boundaries based on the terminal branches of the pulmonary arteries adjacent to the tumor’s safe margin, using cambered surface segmentation techniques. The interdivisional planes of the sublobar and lobar structures, including the vascular roots and passageways, were hollowed out, and a spherical safety margin was created and hollowed around the tumor.

**Figure 2:**
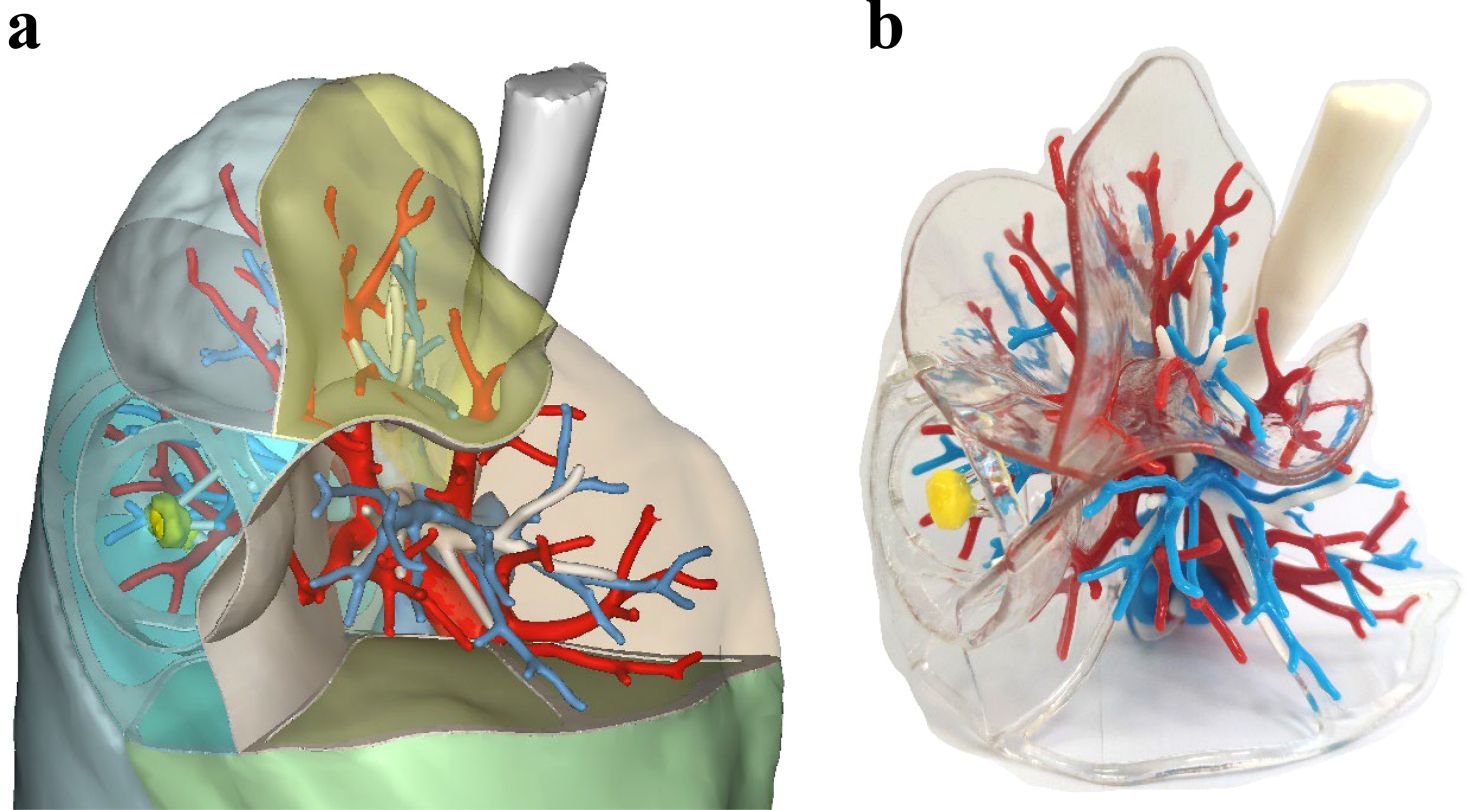
**(a)** 3DV model of early-stage lung cancer in the right upper lobe; **(b)** 3DP model of early-stage lung cancer in the right upper lobe with transparent hollow sublobar boundaries and safe margins, without lung parenchyma. The trachea is represented in white, veins in red, arteries in blue, and the tumor in yellow.

On this basis, the hollowed cambered planes representing the fissures, lobar, sublobar, and safety boundaries were given a 1.5mm thickness for printing. This not only highlighted the tumor’s safe margin and the sublobar boundary affected by the resection but also allowed clear visualization of neighboring blood vessels that needed protection during surgery. The generated STL file of the 3DV model was then imported into Voxel Dance Additive 3.0 preprocessing software (Voxel Dance, China) for model inspection and repair. Finally, the data was sent to a J501Pro 3D printer (Zhuhai Sailner 3D Technology, China) to produce the transparent, hollow 3DP model with sublobar boundaries but without lung parenchyma (**Figure 2**).

### Teaching intervention

Both groups of interns received the same teaching content, delivered by a senior thoracic surgeon. The course focused on a patient with early-stage double primary lung cancer in segment S2 of the right upper lobe. It covered lung anatomy, specifically segmental and subsegmental divisions, knowledge of safe margins for early-stage lung cancer, resection range, and related surgical treatment strategies. The two groups then proceeded to further learning and practice using different teaching methods. The 3DV group learned with a 3D visualization model, interacting with the digital model on a computer to study lung anatomy and surgical procedures. In contrast, the 3DP group used a 3DP model featuring transparent, hollow sublobar boundaries and safe margin, with no lung parenchyma, allowing for a more intuitive observation of internal structures and the surgical resection range.

The surgical procedure and navigation are explained as follows: After routine disinfection and draping, a single-port thoracoscopic incision is made at the 4th or 5th intercostal space along the anterior axillary line to explore the pleural cavity for any adhesions. Once safe entry is achieved, the affected lung lobe containing the nodule is identified. Using either the 3DV or 3DP model, the bronchus, arteries, veins, and lung segment requiring resection are determined. Subsequently, the corresponding vessels and bronchi are mobilized and dissected. The intersegmental plane is then observed, followed by segmentation and resection. The differences between the teaching methods for the two groups are shown in **Table 1**.

**Table 1:** The differences between the two model-based teaching methods.

Variables	3DV group	3DP group
Teaching Model	Digital Model	Physical Model
Magnification/Reduction	Yes	No
Display/Hide Features	Yes	No
Hollow design	Yes	Yes
Life-size physical model	No	Yes
Stereoscopic Vision–Haptic Feedback	No	Yes
Surgical Simulation	No	Yes

A theory test was administered to both groups one month before and immediately after the teaching intervention, with a 30-minute time limit. The test consisted of three parts: fill-in-the-blank questions, image recognition, and multiple-choice questions, all focused on thoracic surgery. Additionally, after the final test, all interns completed a questionnaire that rated the advantages of each group’s model on a scale of 1 to 5. The questionnaire assessed the following aspects (1): The convenience of the model (2); The intuitiveness of the model (3); Identification of the lung segment range affected by the safety margin (4); The difficulty level of acquiring knowledge (5); Understanding of the spatial relationships of blood vessels. Participants were also encouraged to provide any suggestions at the end of the survey.

### Statistical analysis

Data analysis was performed using IBM SPSS Statistics for Windows, Version 26.0 (Armonk, NY: IBM Corp.). Continuous variables were expressed as mean ± standard deviation, and independent samples t-tests were used for between-group comparisons. Categorical data were expressed as frequency (%), and the chi-square test was used for group comparisons. All P-values were two-sided, with *P* 0.05 considered statistically significant.

## Results

### Baseline characteristics of the students

A total of 62 clinical medical students were included in this study. The 3DV group consisted of 31 students, with 17 males and 14 females, and an average age of 21.97 ± 1.08 years. The 3DP group also included 31 students, with 16 males and 15 females, and an average age of 22.17 ± 0.93 years. There were no statistically significant differences between the two groups in terms of age or gender (*P* > 0.05) (**Table 2**).

**Table 2:** Comparison of the baseline characteristics.

Variables	3DV	3DP	*P* value
Age(years)	21.97 ± 1.08	22.17 ± 0.93	0.453
Sex			0.799
Male(n,%)	17(54.8%)	16(51.6%)	
Female(n,%)	14(45.2%)	15(48.4%)	

### Theory test scores

**Figure 3:**
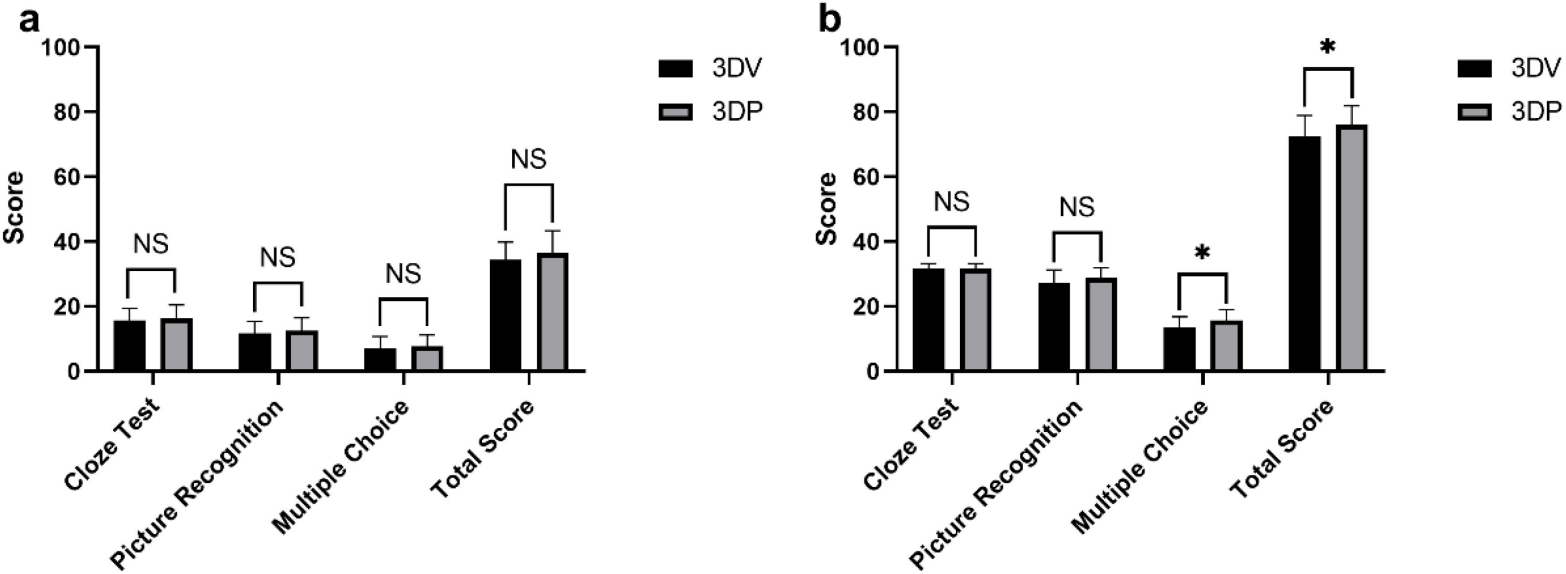
Comparison of test scores between the 3DV (n=31) and 3DP (n=31) groups at two time points. **(a)** Comparison of pre-teaching scores between the two groups; **(b)** Comparison of post-teaching scores between the two groups. * indicates *P* < 0.05, NS: Not Significant.

Before the teaching intervention, there were no significant differences between the 3DV group and the 3DP group in the scores for thoracic surgery knowledge in fill-in-the-blank, image recognition, multiple-choice questions, or total scores (*P* > 0.05) (**Table 3**). After the teaching intervention, the total scores of the 3DP group were significantly higher than those of the 3DV group (*P* = 0.021) (**Table 4**). Specifically, the 3DP group scored significantly higher in multiple-choice questions compared to the 3DV group (*P* = 0.012). Although the scores for fill-in-the-blank and image recognition questions improved after teaching, no significant differences were observed between the two groups (*P* > 0.05) (**Figure 3**).

**Table 3:** Comparison of pre-teaching scores between the two groups.

Pre-teaching Test Scores	3DV group(n=31)	3DP group(n=31)	*P* value
Cloze Test	15.74 ± 3.72	16.39 ± 4.05	0.516
Picture Recognition	11.74 ± 3.57	12.52 ± 3.97	0.422
Multiple Choice	6.97 ± 3.72	7.61 ± 3.63	0.492
Total Score	34.45 ± 5.43	36.52 ± 6.83	0.193

**Table 4:** Comparison of post-teaching scores between the two groups.

Post-teaching Test Scores	3DV group(n=31)	3DP group(n=31)	*P* value
Cloze Test	31.61 ± 1.59	31.74 ± 1.44	0.738
Picture Recognition	27.35 ± 3.88	28.77 ± 3.17	0.120
Multiple Choice	13.42 ± 3.35	15.61 ± 3.32	0.012
Total Score	72.39 ± 6.56	76.13 ± 5.80	0.021

### Questionnaire survey results

After the teaching intervention, each intern was provided with a questionnaire regarding the teaching models, with a 100% response rate. The results (**Table 5**) showed that the 3DV group significantly outperformed the 3DP group in terms of model convenience(*P*<0.001). However, the 3DP group was rated significantly higher than the 3DV group in terms of the ease of knowledge acquisition and understanding of vascular spatial relationships(*P*<0.001). There was no significant difference between the two groups regarding model intuitiveness and recognition of lung segment range influenced by the safety margin. The overall score of the 3DP group is significantly higher than that of the 3DV group(*P*=0.02).

**Table 5:** Comparison of questionnaire results between the two groups.

Assessment items	3DV group(n=31)	3DP group(n=31)	*P* value
The convenience of the model	3.68 ± 0.59	2.94 ± 0.77	<0.001
The intuitiveness of the model	3.26 ± 0.45	3.42 ± 0.50	0.185
Identification of the lung segment range affected by the safety margin	3.13 ± 0.50	3.23 ± 0.56	0.476
The difficulty level of acquiring knowledge	2.94 ± 0.25	3.42 ± 0.50	<0.001
Understanding of the spatial relationships of blood vessels	2.74 ± 0.68	3.97 ± 0.48	<0.001
Total Score	15.74 ± 0.96	17.00 ± 1.47	0.02

## Discussion

The application of 3DP technology in the medical field has rapidly progressed from the research stage to clinical practice, mainly being applied in surgical procedures and clinical education. For clinical interns, understanding complex anatomical pathology and surgical procedures is a critical challenge in medical education (11). 3DP models offer precise, individualized, and life-size physical models that help interns better grasp the spatial relationships between tumors, bronchi, and vascular structures, facilitating the understanding of surgical procedures and intraoperative navigation (12).

In this study, the optimized 3DP model demonstrated three significant advantages in teaching applications. First, during the 3D reconstruction process, hollow interdivisional planes were incorporated into the 3DV model as sublobar boundaries using computer-aided design technology. These boundaries were printed with transparent materials, forming a clear, hollow separation structure in the 3DP model. This design allowed the students to easily distinguish between lung segments and subsegments, facilitating the understanding of the anatomical relationships between the target area and critical lung structures. It addressed the teaching challenges associated with identifying which vessels and bronchi to resect or preserve during sublobar resection. This model not only highlighted the sublobar structures requiring resection based on the tumor location but also allowed for observation of the adjacent structures that needed protection, especially the root of vessels. Second, the 3DP model omitted the printing of lung parenchyma. By displaying the lung lobe contours while avoiding issues like glare and refraction errors caused by transparent resin, the model provided a clearer visualization of vascular pathways within sublobar structures. Without the obstruction of lung parenchyma, interns could directly touch the vascular structures, enhancing both visual and tactile feedback, which deepened their understanding of vascular locations and helped them learn how to avoid intraoperative vascular injuries. Third, in sublobar resection, determining the correct anatomical plane is crucial, but ensuring an adequate surgical margin is even more important (13). In our model, a hollow spherical safety margin was marked around the tumor, clearly delineating the surgical resection area and the target sublobar divisions. This approach not only helped surgeons intuitively understand the extent of the resection preoperatively but also allowed them to simulate the procedure, thereby improving surgical precision. Through the demonstration using 3DP models, students and surgeons could grasp the techniques for segmental and subsegmental localization and resection. By progressing from the complete lung structure to an in-depth understanding of sublobar anatomy, students could better memorize important vascular structures, enhancing the effectiveness of clinical teaching and shortening the learning curve.

This study found no significant difference in the theoretical test scores between these two groups before the teaching intervention, indicating that these two groups of interns had comparable baseline knowledge. However, after the teaching intervention, the 3DP group performed significantly better than the 3DV group (**Figure 3**). This may be due to the physical nature of the 3DP model, which provided a more intuitive and tangible learning experience, helping students better understand and retain complex anatomical structures and surgical procedures. In the testing design of this study, the fill-in-the-blank and image recognition questions primarily assessed students’ basic understanding of lung segment naming, location, and simple spatial relationships, with a focus solely on the right upper lobe, which is a relatively simple anatomical area. Therefore, the overall difficulty is relatively low, making it challenging to demonstrate significant differences between teaching methods in terms of foundational knowledge. In contrast, the multiple-choice questions encompassed more complex and reasoning-based scenarios, addressing deeper understanding of safe margins, variant vascular anatomy, and sub-lobectomy strategies. As a result, these questions were more effective in differentiating students based on their spatial imagination and clinical application abilities. Consequently, the teaching advantage of 3DP models became more apparent in the multiple-choice questions. This indirectly suggests that providing highly realistic 3D models in more challenging clinical scenarios may help students develop a deeper spatial cognition and enhance their comprehensive analytical skills. Several authors have already demonstrated the educational benefits of using 3DP anatomical models in other kinds of medical education (14–17).

The score differences between the 3DV and 3DP groups suggest that the surgery-optimized 3DP model holds greater value in early-stage lung cancer clinical education. As shown in **Table 4**, the 3DV group demonstrated a significant advantage in terms of model convenience. Unlike the physical 3DP model, the 3DV model can be stored on various electronic devices, making it easier to carry. Moreover, due to the replicability of digital models, each student can access the 3DV model on their phone or personal computer without needing to share a physical model. However, with the increasing availability and decreasing cost of 3DP, if every student could have their own physical model in the future, this advantage of the 3DV model might become less significant.

In contrast, the 3DP model was significantly better than the 3DV model in terms of the ease of knowledge acquisition and understanding of vascular spatial relationships. This was mainly due to its physical characteristics, which provided intuitive 3D perception and tactile feedback, enhancing learners’ comprehension of complex anatomical structures. Additionally, the 3DP model facilitated hands-on interaction and operational learning during the teaching process, improving the students’ spatial cognition. These advantages made the 3DP model more effective for teaching complex anatomy compared to the 3DV model. However, there was no significant difference between the two groups in terms of model intuitiveness and understanding of the lung segment range influenced by the safe margin.

The essence of this surgery-optimized 3DP innovation lies in its innovative design of surgical elements, allowing users to fully grasp surgical information—information that would otherwise require a series of interactive steps, such as showing, hiding, or adjusting transparency on a 3DV model—without needing complex interactions. This not only compensates for the lack of interactivity in the 3DP model but also fully leverages the 3DP model’s synergistic visual and tactile feedback.

With the development of medical-engineering interdisciplinary technology, the integration of 3DP and surgery will become even closer. The ability of 3DP to produce personalized models with complex spatial structures makes it especially suitable for application in the surgical field (18). Our study explored the application of 3DP technology in the clinical education of thoracoscopic sublobar resection. The 3DP model’s high-fidelity anatomical representation and intuitive 3D display greatly enhanced the teaching experience and the students’ surgical simulation practice. However, it is undeniable that the cost, production time, and technical complexity of 3DP are limiting factors for its widespread application (19). In this study, the cost of the 3DP model used is approximately ¥1500, based on current common material costs and printing expenses; however, the exact cost may vary depending on factors such as the model’s size, complexity, and design precision. In addition, the investment in equipment and technology primarily involves three-dimensional reconstruction. Although the initial investment incurs a certain expense, it is a one-time cost and is expected to decrease further as the technology becomes more widespread.

Future research could further explore the long-term retention of knowledge and practical skills among students using these teaching methods and evaluate their applicability at different stages of learning. A combination of 3DV and 3DP technologies may further enhance teaching outcomes. For example, students could first gain an overall understanding through 3DV technology, followed by detailed study and hands-on practice with a 3DP model, addressing the lack of practical operation opportunities in traditional teaching methods. As a standalone model, the surgery-optimized 3DP model addresses many of the previous shortcomings of 3DP models. Its sophisticated design for surgical optimization allows it to more effectively support teaching compared to the 3DV model. This also lays a solid foundation for the future use of such 3DP models in preoperative planning and intraoperative navigation.

## Conclusion

The application of the innovative 3DP model in the clinical teaching of sublobar resection for lung cancer is both feasible and effective. This novel teaching method significantly enhances students’ spatial understanding of anatomical structures and their mastery of surgical techniques, providing a highly intuitive and immersive learning and operational experience. Its wide application potential not only improves teaching outcomes but also offers new avenues for medical education. Furthermore, it is worth exploring its broader clinical applications, including its potential use in surgical navigation.

## Data Availability

The raw data supporting the conclusions of this article will be made available by the authors, without undue reservation.
