# Supplementary material for: From virtual to reality: application of a novel 3D printing hollow model for early-stage lung cancer in the clinical teaching of thoracoscopic sublobar resection
Source: Front Oncol. 2025 May 27;15:1526592. doi: 10.3389/fonc.2025.1526592 (PMC12149187; doi:10.3389/fonc.2025.1526592)
Supplement: Supplementary file 1 [file Table1.docx]

**Group: Age: Sex: ID: Scores:**

**Full score: 100 points**

**Cloze Test (32’)**

1. The lung is generally divided into ________lobes and ________ segments. （Please fill in the number）

2. The left lung upper lobe has ________ segments. （Please fill in the number）

3. The right lung upper lobe has ________ segments. （Please fill in the number）

4. The left lung has a total of ________ segments. （Please fill in the number）

5. The right lung has a total of ________ segments. （Please fill in the number）

6. The structure closely associated with the bronchus is ________.

7. The pulmonary artery delivers ________ blood to the lungs.

8. S1 belongs to the right lung ________ lobe.

**Picture Recognition (40’)**

1. Which lung lobe is indicated by the arrow, and how many segments does this lobe contain?


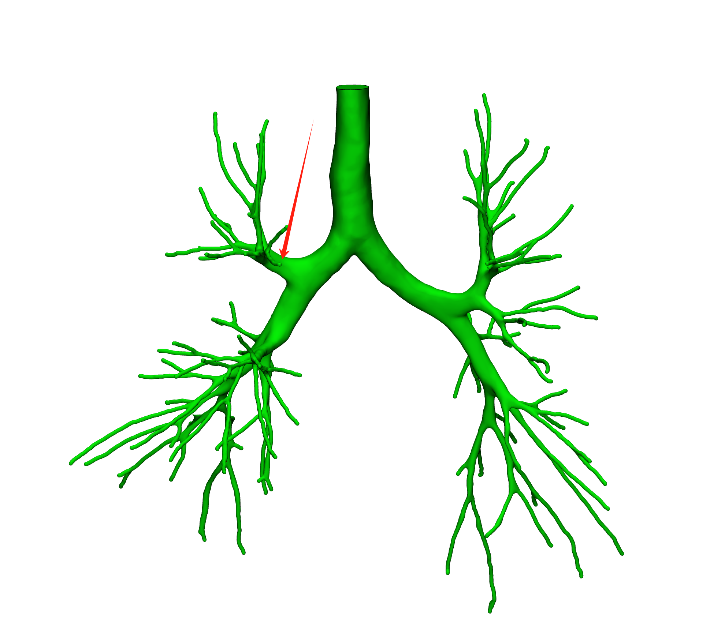


2. Which lung segment is indicated by the arrow in the image? Which segment is located in front of it?


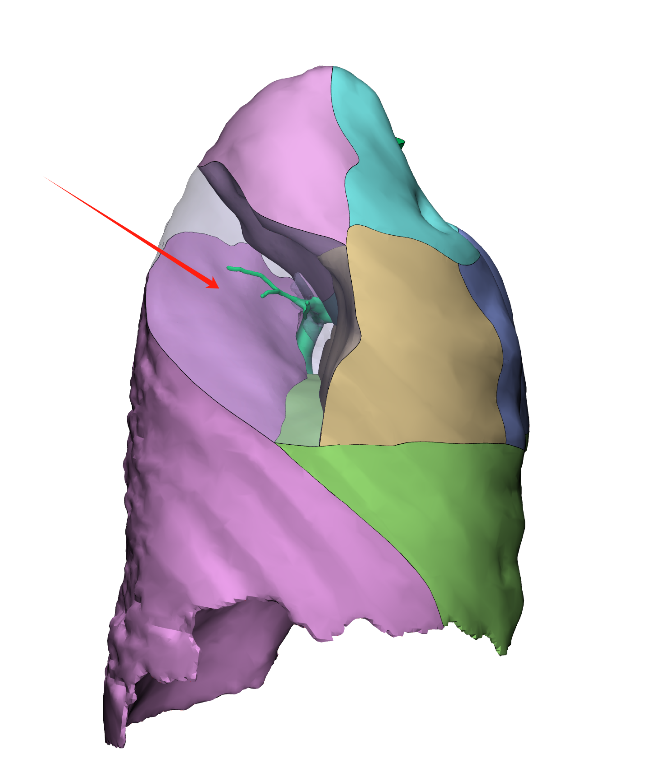


3. In which segment is the tumor located in the image? Which bronchus(bronchi) need to be resected?


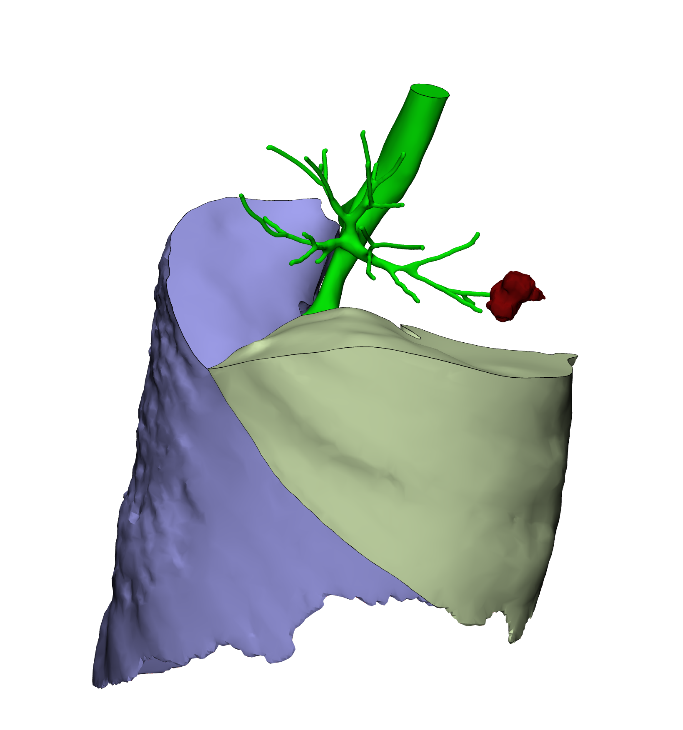


4. Which bronchus (bronchi) in the image will be affected by the safety boundary? Which segments need to be resected?


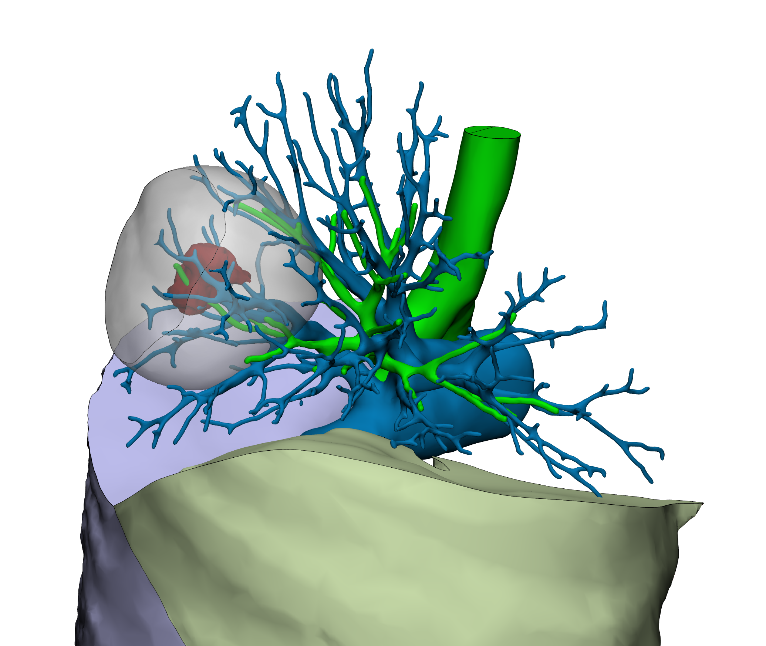


5. Which subsegments are included within the safety boundary indicated by the arrow in the image? If resection is needed, which arteries and veins need to be ligated?


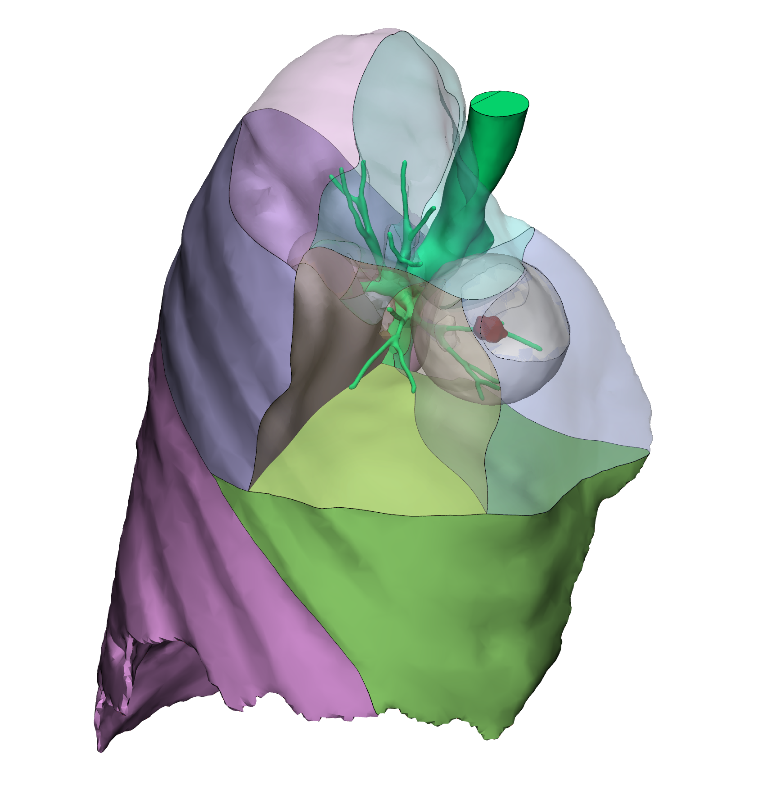


**Multiple Choice (28’)**

1. In surgery related to the right upper lobe of the lung, a mixed ground-glass nodule is located in S3 of the right upper lobe, with a Tumor solid component ratio （CTR）of 0.7 and a diameter of 18mm near the horizontal fissure. After resecting S3, intraoperative frozen section pathology shows early-stage cancer, with a 15mm distance from the tumor margin to the surgical margin. What is the most appropriate next step?

A. No further lung tissue resection, continue with other steps of the surgery. B. Further expand the resection margin, at least 5mm.

C. Terminate the surgery and proceed with radiation therapy.

D. Consider resecting the remaining right upper lobe.

2. In sub-lobar resection surgery of the right upper lobe, the main factor that does not contribute to determining the safe surgical margin is:

A. Tumor size and location

B. Tumor solid component ratio (CTR)

C. Intraoperative rapid pathology result of the nodule type

D. Pulmonary adhesions

3. In pulmonary nodule resection surgery, which of the following are common types of resection margins? (Multiple answers may be correct)

A. Combined subsegmenctomy

B. Combined segmentectomy

C. Segmentectomy

D. Wedge resection

E. Pleural resection

4. Right upper lobe V1a runs between ______ and ______?

A. S1a; S1b

B. S1b; S3b

C. S2b; S3a

D. S1a; S2a

5. Right upper lobe V1b runs between ______ and ______?

A. S1a; S1b

B. S1b; S3b

C. S2a; S2b

D. S3a; S3b

6. What runs between S2a and S2b of the right upper lobe?

A. V2a

B. V2b

C. V2c

D. V1I

7. What variation is observed in the bronchus in the image?


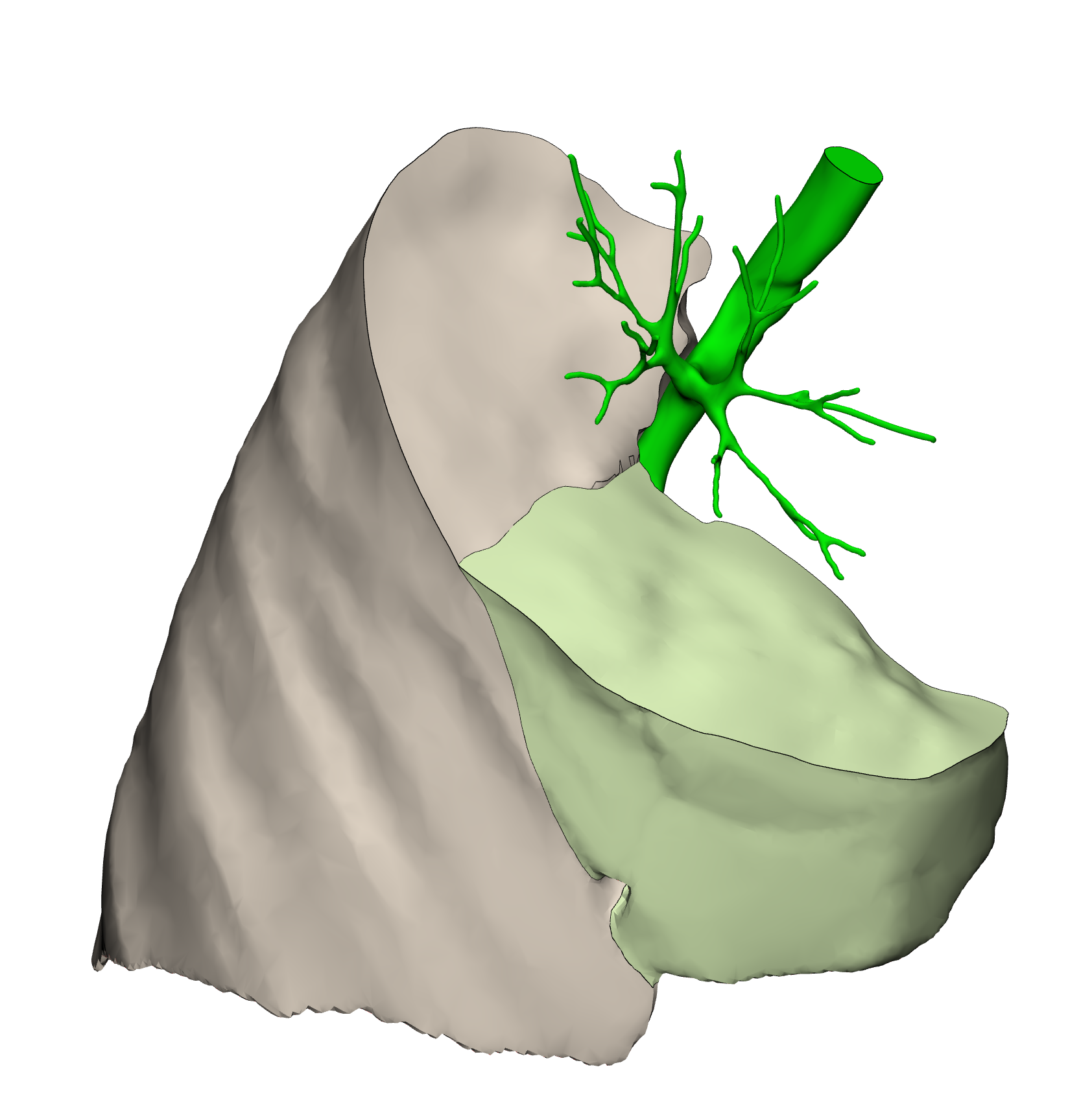


A. B1 is absent, leaving only B2 and B3. S2 and S3 are abnormally enlarged.

B. B1 originates abnormally from B2, and S1 is smaller than normal.

C. B1 has a bifurcation anomaly, with B1a and B1b originating from the B2 and B3 trunks, respectively.

D. B1 originates abnormally from B3, and S1 is smaller than normal.
